# Standardization of presurgical language fMRI in Greek population: Mapping of six critical regions

**DOI:** 10.1002/brb3.2609

**Published:** 2022-05-19

**Authors:** Kostakis Gkiatis, Kyriakos Garganis, Christopher F. Benjamin, Irene Karanasiou, Nikolaos Kondylidis, Jean Harushukuri, George K. Matsopoulos

**Affiliations:** ^1^ School of Electrical and Computer Engineering National Technical University of Athens Athens Greece; ^2^ Epilepsy Monitoring Unit St. Luke's Hospital Thessaloniki Greece; ^3^ Department of Neurology Comprehensive Epilepsy Center Yale School of Medicine New Haven Connecticut USA; ^4^ Department of Neurosurgery Yale School of Medicine New Haven Connecticut USA; ^5^ Radiological Unit St. Luke's Hospital Thessaloniki Greece

**Keywords:** fMRI, Greek, language, neurology, neuropsychology, neurosurgery, presurgical

## Abstract

**Background:**

Mapping the language system has been crucial in presurgical evaluation especially when the area to be resected is near relevant eloquent cortex. Functional magnetic resonance imaging (fMRI) proved to be a noninvasive alternative of Wada test that can account not only for language lateralization but also for localization when appropriate tasks and MRI sequences are being used. The tasks utilized during the fMRI acquisition are playing a crucial role as to which areas will be activated. Recent studies demonstrated that key language regions exist outside the classical model of “Wernicke–Lichtheim–Geschwind,” but sensitive tasks must take place in order to be revealed. On top of that, the tasks should be in mother tongue for appropriate language mapping to be possible.

**Methods:**

For that reason, in this study, we adopted an English protocol that can reveal six language critical regions even in clinical setups and we translated it into Greek to prove its efficacy in Greek population. Twenty healthy right‐handed volunteers were recruited and performed the fMRI acquisition in a standardized manner.

**Results:**

Results demonstrated that all six language critical regions were activated in all subjects as well as the group mean map. Furthermore, activations were found in the thalamus, the caudate, and the contralateral cerebellum.

**Conclusion:**

In this study, we standardized an fMRI protocol in Greek and proved that it can reliably activate six language critical regions. We have validated its efficacy for presurgical language mapping in Greek patients capable to be adopted in clinical setup.

## Graphical abstract

fMRI tasks should be performed in mother tongue for activations to be reliable. As such, we translated an English fMRI protocol and standardized the procedure to validate its efficacy in Greek population. By performing the task in 20 healthy volunteers, we proved that the protocol can reliably activate all six language critical regions in Greek even in clinical setup for presurgical evaluation purposes.

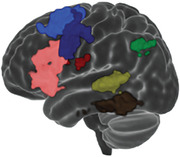

## 1 INTRODUCTION

Functional magnetic resonance imaging (fMRI) identifies the brain areas activated during a specific task performed by subjects during acquisition. By alternating periods of performing the task with control periods, statistically significant differences related to task and/or stimuli compared with the control periods may be detected in the respective brain areas (Singleton, 2009). fMRI has gained widespread use in neurosurgery, especially for language network mapping when the area of anticipated resection is located close to relevant cortex (Benjamin et al., 2020; Brennan & Hadley, 2009; Filippi, 2016).

### 1.1 The language system

Since the early days, epilepsy surgeries, especially in temporal lobe epilepsy, as well as tumor removals have been a key motivation for identifying language lateralization and within‐hemisphere localization in order to avoid unpredictable language deficits (Galaburda & Geschwind, 1980; Loring et al., 1990).

#### 1.1.1 Mapping of six language critical regions

In clinical settings, the main focus is to map the classic model of Wernicke–Lichtheim–Geschwind even if formidable progress in the field was achieved in the past decades (Tremblay & Dick, 2016). A recent study by Benjamin et al. (2017) showed that it is possible in routine clinical setup to map the language system that involves these six language critical regions. In their study, they used three different tasks combined with a methodology that included conjunction maps to subtract the stimulus‐specific activations and a varying thresholding among tasks. All six language critical regions were localized in most of their subjects. This study provides evidence that experienced clinicians using their protocol may generate language maps that incorporate a wider language system than that of the Wernicke–Lichtheim–Geschwind model.

##### Broca's area

In 1861, Paul Broca demonstrated that inferior frontal gyrus (IFG) in the left hemisphere is responsible for language articulation. According to the author's definition, Broca's area is located in the posterior third of the IFG. Besides pars opercularis and pars triangularis, several clinical and fMRI studies suggest that pars orbitalis, part of the middle frontal gyrus (MFG), frontal operculum, and anterior insula, are also included in “Broca's Complex” (Desai et al., 2006; Longe et al., 2007; Połczyńska et al., 2017). Pathology in these areas can result in agrammatism, anarthria, low verbal output, and language production deficits (Alfredo Ardila, 2021; Donnan et al., 1999; Schäffler et al., 1996).

##### Wernicke's area

Later, in 1874, Carl Wernicke demonstrated that superior temporal gyrus (STG) in the left hemisphere is responsible for language comprehension (Wernicke, 1874). While for Broca's area there is a relative consensus for the anatomical landmarks that define it, no such consensus exists for the Wernicke's area (Tremblay & Dick, 2016). Most authors define as Wernicke's area the posterior aspect of the STG, while others may extend it to the supramarginal gyrus, the middle temporal gyrus (MTG), in more anterior STG, or even in the inferior temporal gyrus (ITG) (Binder, 2017). While the author himself thought that this area was associated with language comprehension (Wernicke, 1874), more recent lesion and fMRI studies have questioned this association, providing evidence that pathology in this area can cause conduction aphasia with the comprehension remaining intact (Ardila et al., 2016a; Binder, 2017; Turken & Dronkers, 2011). At present, several clinical language mapping studies are parcellating Wernicke's area into an inferior and a superior aspect. The inferior portion is involved in auditory language comprehension and it seems to have bilateral representation. The superior portion subserves word recognition, phoneme perception, and semantic memory modulation (Acheson et al., 2011; Benjamin et al., 2017; Binder, 2015, 2017).

First Lichtheim (1885) then later Geschwind (1970) put that into perspective to create a language model known as the “Wernicke–Lichtheim–Geschwind” model. Even if this is a very simplified model describing two distinct regions, Broca and Wernicke’ areas, connected with a single fiber tract, the arcuate fasciculus, it is a language model still widely in use (Tremblay & Dick, 2016). As we move past this “classic model” to a broader model, there is immense evidence mainly from fMRI and electrocortical studies that other regions participate in language functions as well (Alfredo et al., 2016b).

##### Exner's area

Sigmund Exner first draw attention to the existence of a handwriting region centered in the caudal MFG (Exner, 1881). Since then, many fMRI‐based language studies have found activation in this region, though they rarely call it Exner's area. Many authors prefer to categorize it as a region in the “Broca complex” or as “graphemic motor frontal area” (Alfredo et al., 2016b; Black et al., 2019). Stimulation studies have connected Exner's area with the ability to write (Lubrano et al., 2004). Recent studies indicate that lesions of this area result in speech articulation, semantic processing, or pure apraxia. On the other hand, positive electrocortical stimulation responses in this area revealed hesitation and phonemic errors as well as semantic paraphasia and anomia demonstrating the wide range of language processing that it is involved in (Chang et al., 2020; Hazem et al., 2021). All this convergent evidence shows not only the existence of the Exner's area, but also the key role it plays in numerous language functions.

##### The supplementary motor area

The supplementary motor area (SMA) is another region that is included in “Broca's Complex” even if it is located in the mesial aspect of the superior frontal gyrus (Alfredo Ardila, 2021; Desai et al., 2006). The existence of the frontal aslant tract supports this claim as it connects the IFG, that is, Broca's area, with SMA (Dragoy et al., 2020). Stimulation studies of this area have shown that it can impair sentence completion or delay sentence completion, provoke reading difficulties, and in some cases even display complete speech arrest (Dragoy et al., 2020; Fujii et al., 2015; Rozanski et al., 2015). A recent study of 12 patients undergoing surgery pinpointed the postoperative outcome in the vicinity of frontal aslant tract (Dragoy et al., 2020). They showed that in the acute phase, seven patients demonstrated various deficits, from mild deterioration to severe aphasia. However, in the late period testing (4–14 months from operation), only one subject still presented severe deficits in spontaneous speech, whereas the others have considerably improved and managed to return to normal or close to normal levels. Another study backed this up indicating that the left‐dominant SMA migrates to its homolog region in the nondamaged right hemisphere (Chivukula et al., 2018). These results demonstrate the overall, transient nature of the most severe SMA lesion‐induced dysphasic, although much controversy still remains in this subject.

##### Angular gyrus

As the field has moved beyond the classic Wernicke–Lichtheim–Geschwind model, the angular gyrus (AG) is one of the regions that is being introduced as belonging to the “Wernicke's system” (Alfredo et al., 2016b). Even if aspects of AG function were first highlighted by Gerstmann (1940) showing that if damaged agraphia, acalculia, left and right disorientation, and finger agnosia may occur, there is still a lack of understanding of the exact functions supported by this area. Moreover, most studies report that their patients displayed a subset of these symptoms rather than all of them (Alfredo Ardila et al., 2000; Gold et al., 1995). A study by Price et al. (2015) showed that AG plays a key role in processing of meaningful word combination. Electrocortical stimulation of this area has demonstrated that all subjects presented symptoms in the spectrum of Gerstmann's syndrome. However, there was considerable variability with regard of the exact anatomical areas producing these effects with some cases being located in posterior‐inferior aspect and in other cases being located in more superior sites (Roux et al., 2003).

##### Basal temporal language area

Another important region for language function, as many studies with different modalities are suggesting, is the basal temporal language area (BTLA) (Binder et al., 2020; Mani et al., 2008; Papathanassiou et al., 2000). BTLA is mainly located in the fusiform gyrus, 3–7 cm from the temporal tip (Luders et al., 1991), though most studies suggest that its exact location may vary in individuals making its preoperative mapping crucial (Ojemann, 1979; Steinmetz & Seitz, 1991). Damage in or resection of this region has been associated with word‐finding difficulties, picture naming decline, and semantic paraphasia (Antonucci et al., 2004; Foundas et al., 1998; Kraft et al., 2014). Electrical stimulation studies of BTLA report symptoms such as pure alexia and reading difficulties (Enatsu et al., 2017; Mani et al., 2008). A recent voxel‐based lesion‐symptom study by Binder et al. (2020) provided evidence that BTLA, specifically fusiform gyrus and the adjacent ITG in the left hemisphere, is associated with naming decline when resected.

#### 1.1.2 The language system in Greek

In Greek language, only a handful of fMRIs published papers exists. Malogiannis et al. (2003) performed an auditory discrimination fMRI task in six healthy subjects and they were able to activate Wernicke's and Broca's areas, though not reliably in all subjects. In an attempt to extract the language network from resting‐state fMRI (rs‐fMRI) in 29 patients with left IFG tumor, Liouta et al. (2019) found activations in Broca's area. Kokkinos et al. (2021) exploring the feasibility of language task‐fMRI in patients discovered that it can be applied even to severe tumor cases activating both Broca's and Wernicke's regions independent of covariates such as age, gender, handedness, and education level. A study not limited to the Wernicke–Lichtheim–Geschwind was made by Protopapas et al. (2016). A lexical decision task was performed by 44 healthy volunteers and they were able to activate the MFG, the AG, and the fusiform gyrus apart from Broca's and Wernicke's areas, though their focus was to identify orthographic and graphophonemic systems.

#### 1.1.3 The present study

The present study focuses on the evaluation of the fMRI approach of Benjamin et al. (2017) to map these six language critical regions in Greek by developing a standardized form of their protocol for native Greek‐speaking adults. As in the original article the model of the language system is based almost exclusively on studies of patients speaking English, the generalization of these results to other languages remains unclear. The protocol was translated and adapted to the Greek language and it was evaluated in a group of 20 healthy volunteers free from any neurological disorder. The recordings were performed using professional equipment to account for the noisy conditions that exist in an MRI room. We hypothesized that the same language system will be revealed in our cohort. We also hypothesized that the six language critical regions will be found in most of the subjects in the individual‐level analysis.

## 2 MATERIAL AND METHODS

### 2.1 Subjects

Healthy controls were recruited for evaluation purposes of the language protocol that has been translated in the Greek language. Data were acquired from September 2018 through July 2020 in St. Luke's Hospital, Thessaloniki, Greece and all subjects gave their consent to participate in the study prior to their inclusion. Inclusion criteria in the study were as follows: (a) Greek native speaker, (b) aged 18–45, (c) right handed (self‐reported), (d) normal or corrected‐to‐normal vision, (e) no history of neurological disease, and (f) no history of drug abuse. Under these criteria, 20 volunteers participated in the study with a mean age of 31.6 years (±7.4), including 11 females. Three of the subjects (two females) were also fluent in German and one female was fluent in Dutch. The mean education time in years was 16 (±2.2). Handedness was further assessed with the Edinburgh Handedness Inventory—Short Form (Veale, 2014) and all subjects were found right handed (mean laterality quotient (LQ): 93.125 ± 11.81; range: 62.5–100).

### 2.2 Language fMRI protocol

**FIGURE 1:**
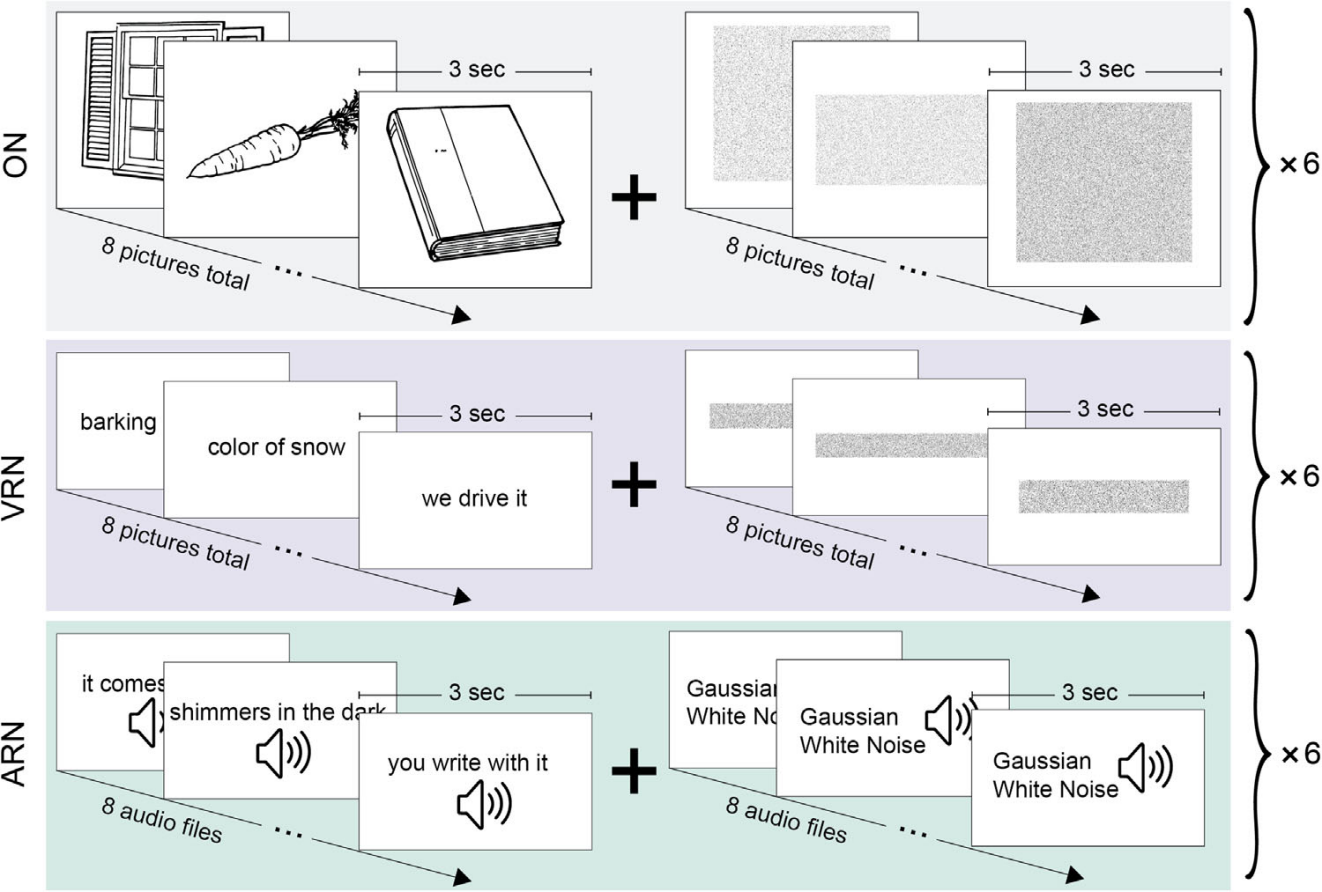
Language fMRI protocol

Three lexicosemantic tasks, as shown in Figure 1, were performed in the Greek language by all subjects and are being evaluated in the current study. All tasks were block designs of 24 s of task period and 24 s of control period alternating six times for a total of 4 min and 48 s acquisition time for each task. More specifically, tasks were (a) Object Naming (ON) (Bookheimer et al., 1995). Task period consisted of alternating line drawn objects and subjects were asked to silently name each object and an action they could perform with it. Control period consisted of alternating scrambled images with similar properties as the pictures shown in task period and subjects were instructed to silently scan the images with their eyes without having any particular thought. Pictures were alternating every 3 s. (b) Verbal Responsive Naming (VRN) (Gaillard et al., 2002, 2001, 2004). In the task period, a word written description of a concrete noun or adjective was presented to the subject and was asked to read the description and think of the single‐word name of the object. In the control period, a rectangular oblong scrambled image with similar properties as the descriptions in the task period was presented and subjects were asked to silently move their eyes from left to right (as in reading) without moving their head. Both task and control pictures were alternating every 3 s. (c) Auditory Responsive Naming (ARN) (Bookheimer et al., 1997; Gaillard et al., 2004). During the task period, a word description of a concrete noun or adjective was presented through pneumatic headphones to the subjects and they were asked to silently think of the one‐word name of the object being described. During the control period, a Gaussian noise with similar properties with the sounds in the task period was presented and subjects were instructed to silently listen to the tone without losing their attention or having any particular thought. During ARN, subjects had their eyes closed. Both task and control stimuli were alternating every 3 s. More details for the tasks can be found in Benjamin et al. (2017).

All subjects undertook a 20‐min prescan training with items different than the ones presented in the scanner in order to familiarize with the tasks prior to any acquisition. Tasks were presented with the same order (ON–VRN–ARN) in all subjects. During the scanning session and before each task, instructions were presented to the subject for the upcoming task and they were asked if they remember their tasks to ensure compliance. In the beginning of each task, the stimuli presentation was initiated after three fMRI volumes/pulses for scanner stability purposes.

Differences from the English validated tasks from Benjamin et al. (2017) are the control period as in that study rest has been chosen for control, while we chose to show scrambled images or white gaussian noise for control. That choice was made to account for activations due to stimuli itself as the creators of the tasks have performed (Bookheimer et al., 1995; Gaillard et al., 2004, 2001). Another difference are the pictures in the task period as they are different from the ones used in the English version. That choice was mandatory as the pictures in that version are copyrighted and we could not use them, though the essence of the tasks has not been affected from that as the objects shown were the same.

Both English and Greek versions of the tasks as well as Arabic‐Egyptian, Cantonese, Farsi, German, Hindi, Italian, Korean, Polish, Portuguese (European), Punjabi, Spanish (South American; European), and Turkish versions are freely available for download at www.cogneuro.net/omfmri.

### 2.3 MRI setup and acquisition

All tasks were run on Neurobehavioral Systems’ Presentation (https://www.neurobs.com/). The delivery of stimulus was synchronized with the pulse of the MRI scanner for accuracy and standardization purposes. During the acquisition, each subject's head was carefully placed and restricted with foams in the coil. Pneumatic headphones were used for insulation without any music during rs‐fMRI, ON, and VRN and for stimuli presentation during ARN. Pictures and description were shown through a projector placed outside the scanner room on a screen that was at the bottom of the MRI table. A mirror was attached in the head coil and adjusted prior to acquisition for the subject to be able to see the screen properly. The procedure started with a 4‐min field mapping with the appropriate sequences following a 15‐min rs‐fMRI sequence. After that, the three tasks were performed in the same order (ON–VRN–ARN) for all subjects. Following, T1‐weighted and T2‐weighted images of high resolution were obtained for registration, segmentation, and parcellation purposes.

MRI acquisitions were performed in St Luke's Hospital, Thessaloniki, Greece, in an upgraded Siemens Avanto FIT 1.5T MRI scanner (Siemens Healthineers, Erlangen, Germany). Head and Neck coil is a standard Siemens 20‐channel coil with Simultaneous Multi‐Slice (SMS) capabilities. fMRI was conducted using an echoplanar imaging (EPI) sequence with full brain coverage and the following parameters: multiband factor 4, TR (repetition time) 1700 ms, TE (echo time) 50 ms, flip angle 84°, FoV (field‐of‐view) 204 × 204 × 120 mm^3^, voxel size 2 × 2 × 2 mm^3^, matrix 102 × 102 voxels. The same fMRI protocol was used for task as well as rest fMRI with 177 and 530 measurements, respectively. Field mapping was performed with a standard Siemens field mapping sequence, matching the parameters of the fMRI sequence and FoV high enough to cover the whole head: TR: 1010 ms, TE: 4.76 and 9.52 ms, flip angle 60°, voxel size 2 × 2 × 2 mm^3^, FoV 228 × 228 × 170 mm^3^. A three‐dimensional T1‐weighted image with a Magnetization‐Prepared Rapid Gradient‐Echo (MPRAGE) sequence was acquired with the following parameters: GeneRalized Autocalibrating Partially Parallel Acquisitions (GRAPPA) factor 2, TR 2200 ms, TE 2.97 ms, TI (inversion time) 900 ms, flip angle 8°, FoV 250 × 250 × 192 mm^3^, matrix 256 × 256 voxels, voxel size 1 × 1 × 1 mm^3^, axial acquisition. A three‐dimensional T2‐weighted image was also acquired with the following parameters: GRAPPA factor 2, TR 5000 ms, TE 335 ms, TI 1800 ms, FoV 260 × 252 × 176 mm^3^, matrix 256 × 248 voxels, voxel size 1 × 1 × 1 mm^3^, sagittal interleaved acquisition. Data are not publicly available.

### 2.4 Data preprocessing

For the preprocessing and analysis of the dataset, FMRIB's Software Library (FSL; v 6.0.1; https://www.fMRIb.ox.ac.uk/fsl) was utilized (Jenkinson et al., 2012). Data were anonymized prior to any analysis. Since the data consisted of task‐based fMRI, they were minimally preprocessed to avoid introducing unnecessary interpolations and biases. All subjects were separately analyzed with the same pipeline as follows. First, T1‐weighted anatomical images were skull‐stripped using optiBET script, which has been extensively tested in 70 patients’ brain and has outperformed all other tools (Lutkenhoff et al., 2014), for registration purposes. In the fMRI data, the first three volumes were discarded for signal stabilization purposes as well as the last four volumes as the task had been completed. The middle image of the remaining fMRI sequence was used as a template for further analysis. Motion correction was performed to the functional data (MCFLIRT) by registering all images to the template using rigid body transformation with six degrees of freedom (Jenkinson et al., 2002). B0 scanner field inhomogeneities were estimated using the field map sequences and, after registration of the maps to the template image, data were corrected accordingly (Jenkinson & Smith, 2001). Brain extraction of the fMRI was performed in an automated way as implemented in FEAT tool. Spatial smoothing with a Gaussian kernel of 4 mm full width at half maximum (FWHM) was applied to the data to ensure high SNR and at the same time smooth any high peak in the image. In order to ensure valid statistical analysis, grand‐mean intensity normalization was applied to the entire fMRI sequence and a high‐pass filter matching the task sequence period (50 s) was determined. Afterward, registration of the template to the T1‐weighted image was performed with the highly adopted Boundary‐Based Registration (BBR) algorithm which, after applying the extensively used rigid body transformation, performs slight corrections according to the white‐gray matter boundaries (Greve & Fischl, 2009). The MNI152 brain with 2 mm resolution was used as a template for the group analysis. Registration of the T1‐weighted image to the MNI152 template was performed in two steps. In the first step, a linear rigid transformation with 12 degrees of freedom was employed. In the second step, this transformation was utilized as initialization for the nonlinear registration and the warp field estimation of the T1‐weighted to the MNI152 template (Smith et al., 2004; Woolrich et al., 2009).

### 2.5 Statistical analysis

Statistical analysis was performed in two levels. The first level refers to the subject‐specific statistics, while the second level is the group analysis. For each subject, a single language map was created by two experienced fMRI language mapping clinicians, KG and KN.

#### 2.5.1 First‐level analysis

At this level, there were three fMRI datasets for each subject making a total of 60 fMRI datasets. After preprocessing steps, white Gaussian noise was added to each fMRI dataset in order to ensure independence among voxels’ timeseries required for valid statistical analysis. A General Linear Model (GLM) analysis was performed at this stage. The timing and duration of each task and control period after convolving with a Gamma function (std: 3 s; mean lag; 6 s) and applying a high‐pass filter at 50 seconds was used as regressor of interest. The temporal derivative of the task regressor was included to account for slice time differences and differences in hemodynamic responses in different parts of the brain; the six motion parameters were also included as regressors of no interest (Woolrich et al., 2001). It should be noted that the derivatives of the motion parameters were not included, as they would increase the degrees of freedom of the model.

#### 2.5.2 Subject‐specific language map

The procedure that was followed for the creation of the single language map for each subject was in line with the one carried out in the original validation of the tasks (Benjamin et al., 2017). More specifically, the order of the preferred single map was (i) conjunction of all three tasks, (ii) conjunction of ARN and one of VRN or ON, and (iii) a single task. Thresholding was the combination of a fixed threshold of *p* < .05 cluster‐wise and a voxel‐wise threshold that ranged from 2.3 to 3.1 *z*‐score (*p* < .01 to *p* < .0001) in order to avoid introduction of noise clusters while maintaining all six language critical regions if possible. An experienced radiologist and an experienced neurologist, KN and KG, reviewed independently the resulting maps and the entire procedure, each one generating a conjunction map for each subject. The maps were compared and in case of disagreement, the two reviewers concurred on the final choice according to the methodology followed in the English evaluation. Each language map was then parcellated into activation clusters and each cluster was classified structurally to one of the six language critical regions accordingly.

#### 2.5.3 Second‐level analysis

Group analysis was performed with the FMRIB's Local Analysis of Mixed Effects (FLAME1+2) tool that models the variability among subjects allowing inferences to be made about the participating population (Woolrich et al., 2004). Automatic de‐weighting outlier was performed in order to account for possible poor performance in some sessions. Each task was considered a different group and a mean across subjects for each task was created. The voxel‐wise threshold was set to *z*‐score >3.1 and the cluster‐wise threshold was set to *p*‐value <.05 for each group mean task. Subsequently, three group maps were created, one for each task. In order to create one group language map, the conjunction of all three task images was computed. For the comparison maps between all pairs of tasks, the voxel‐wise threshold was set at *p* < .001 family wise error corrected with the Bonferroni method (*p* < .00016 uncorrected; *z*‐score >3.6) in order to control for any false positive due to multiple comparisons, resulting in a total of six maps.

## 3 RESULTS

### 3.1 Subject‐specific language maps

For all subjects, the six critical language areas were successfully reproduced. One subject had a right dominant hemisphere, while all other subjects were left dominant; no subject was found bilateral. In Table 1, the volume of each area in the subject‐specific level as a percentage of the total activation is presented. The table is divided into the dominant and nondominant hemispheres.

**TABLE 1:** The percentage of activated voxels in each region for each subject

		Dominant hemisphere						Nondominant hemisphere						
	Subjects	Broca's area	Wernicke's area	Exner's area	Basal temporal	Angular gyrus	SM area	Broca's area	Wernicke's area	Exner's area	Basal temporal	Angular gyrus	SM area	Laterality index
Area in percentage of activated voxels	Subj_01	35.40	23.07	10.96	8.27	3.94	11.23	2.59	1.88	1.20	0.00	1.45	0.00	0.85736
	Subj_02	18.26	29.68	15.41	10.69	18.11	6.31	0.00	0.53	1.01	0.00	0.00	0.00	0.96921
	Subj_03	28.18	29.31	15.54	8.88	8.66	7.53	0.00	1.53	0.37	0.00	0.00	0.00	0.96199
	Subj_04	20.23	24.37	15.31	20.87	13.98	5.25	0.00	0.00	0.00	0.00	0.00	0.00	1
	Subj_05	30.90	13.84	19.21	13.73	5.06	17.26	0.00	0.00	0.00	0.00	0.00	0.00	1
	Subj_06	17.29	22.04	21.50	17.93	14.00	4.98	0.00	0.31	1.96	0.00	0.00	0.00	0.95459
	Subj_07	33.65	16.06	13.22	3.02	9.04	11.76	0.00	4.46	3.65	0.00	5.13	0.00	0.73503
	Subj_08	19.04	18.84	36.98	5.21	5.45	6.96	0.00	1.21	6.31	0.00	0.00	0.00	0.84961
	Subj_09	20.24	24.13	16.51	3.81	6.38	15.36	2.81	3.21	2.10	5.05	0.39	0.00	−0.72856
	Subj_10	22.71	24.68	17.36	10.67	1.20	12.79	0.00	7.64	2.95	0.00	0.00	0.00	0.78826
	Subj_11	21.85	19.97	21.32	5.39	3.78	11.05	2.18	6.75	6.35	1.36	0.00	0.00	0.66717
	Subj_12	23.81	32.83	8.27	18.95	5.28	10.85	0.00	0.00	0.00	0.00	0.00	0.00	1
	Subj_13	37.29	2.91	22.48	12.73	2.53	10.19	4.14	0.12	3.19	1.10	0.00	3.32	0.76255
	Subj_14	26.21	27.19	19.75	11.12	4.40	11.34	0.00	0.00	0.00	0.00	0.00	0.00	1
	Subj_15	21.54	25.35	28.97	5.88	6.78	11.49	0.00	0.00	0.00	0.00	0.00	0.00	1
	Subj_16	25.33	20.06	24.24	7.55	9.01	9.43	0.00	1.45	2.93	0.00	0.00	0.00	0.9123
	Subj_17	30.01	27.17	11.05	15.33	3.60	7.80	0.00	5.04	0.00	0.00	0.00	0.00	0.8991
	Subj_18	18.96	26.70	25.88	5.76	7.39	4.99	0.00	6.89	3.42	0.00	0.00	0.00	0.79379
	Subj_19	11.70	26.08	19.25	19.96	2.09	18.75	0.00	0.00	2.17	0.00	0.00	0.00	0.95659
	Subj_20	18.89	25.09	16.00	7.77	3.26	18.02	0.00	2.46	0.00	3.99	0.00	4.51	0.78072
	Mean	24.07	22.97	18.96	10.68	6.70	10.67	0.59	2.18	1.88	0.57	0.35	0.39	0.80799
	SD	6.70	6.60	6.71	5.55	4.41	4.22	1.25	2.60	2.02	1.41	1.17	1.22	0.37631

*Note*: This table reveals the variability that can be found in the subject specific level. Only the six language critical regions were taken into account for the extraction of the percentage.

Abbreviation: SM area, supplementary motor area.

**FIGURE 2:**
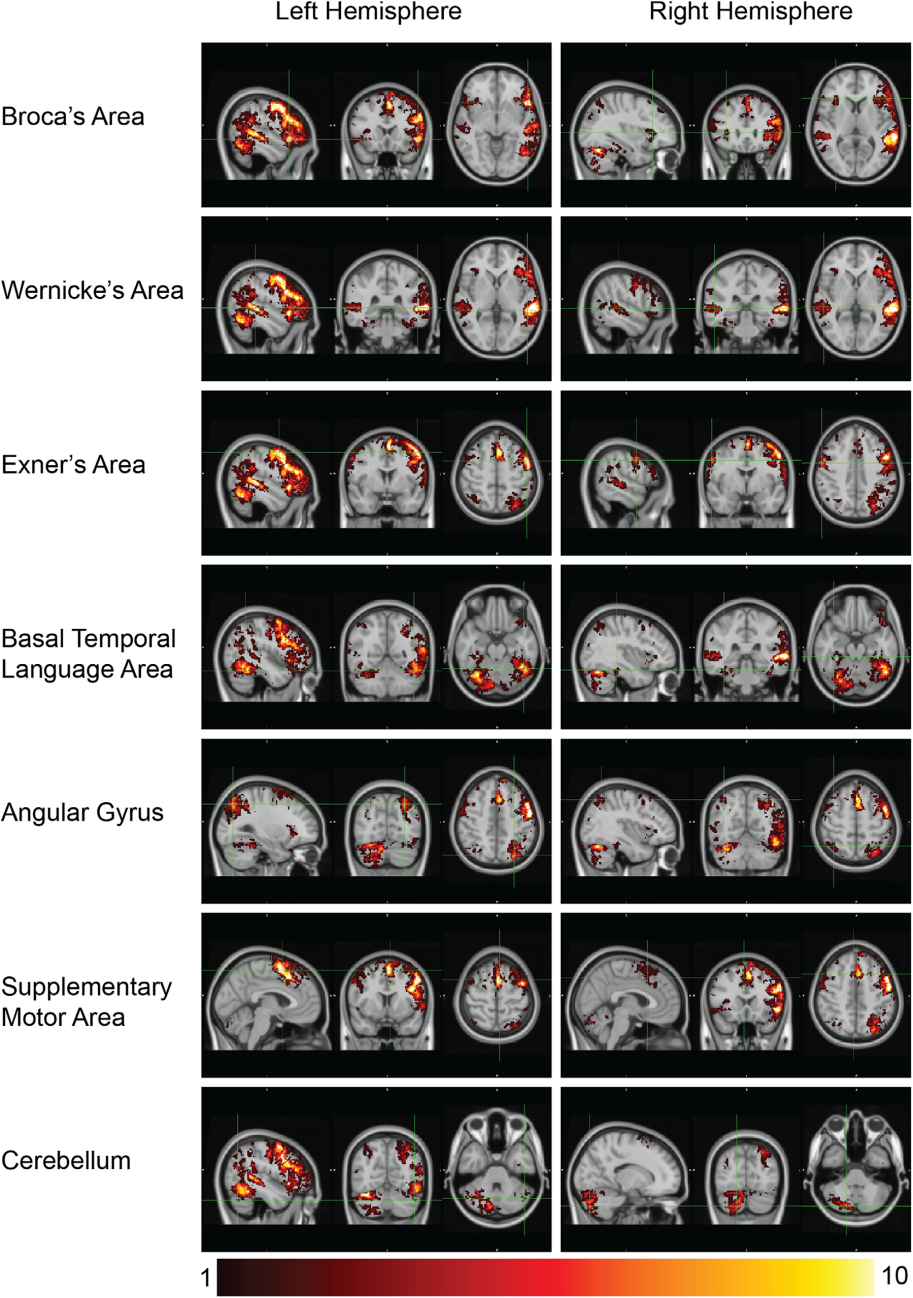
The map of “appearances” showing the frequency with which voxels activated at the single subject level. The map is in MNI152 space and the color bar varies from 1 subject (black) to 10 subjects (white more than 10). This image shows how frequently any given voxel was identified at the single subject level. The form of analysis used can present clinical interest for surgical decision‐making. Images are in radiological orientation

To directly inform clinicians and others using this protocol at the possible extent of each region in the single‐subject level, as in presurgical planning with Greek‐speaking patients, we next sought to identify the typical location of these regions in our sample. This was achieved by directly overlapping the maps for all left language‐dominant subjects to evaluate overlap. The resulting map of “appearances” (Figure 2) shows the number of subjects each voxel was activated to. The six key regions are clearly evident. A large cerebellar cluster was also evident. While all six language critical regions were identified in all subjects, this map shows that there was considerable overlapping in Broca, Wernicke, Exner, and SMA and significant variability in BTLA and AG.

### 3.2 Group‐level language maps

**FIGURE 3:**
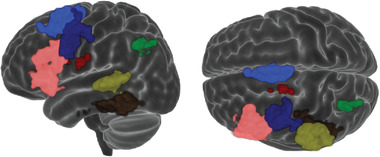
Conjunction map of the three tasks. The regions that were found activated in the left hemisphere of all the three tasks. All six language critical regions are visible as well as a region in thalamus. Pink: Broca. Blue: Exner. Yellow: Wernicke. Light blue: Supplementary motor area. Green: Angular. Brown: Basal temporal language area. Red: Thalamus

**FIGURE 4:**
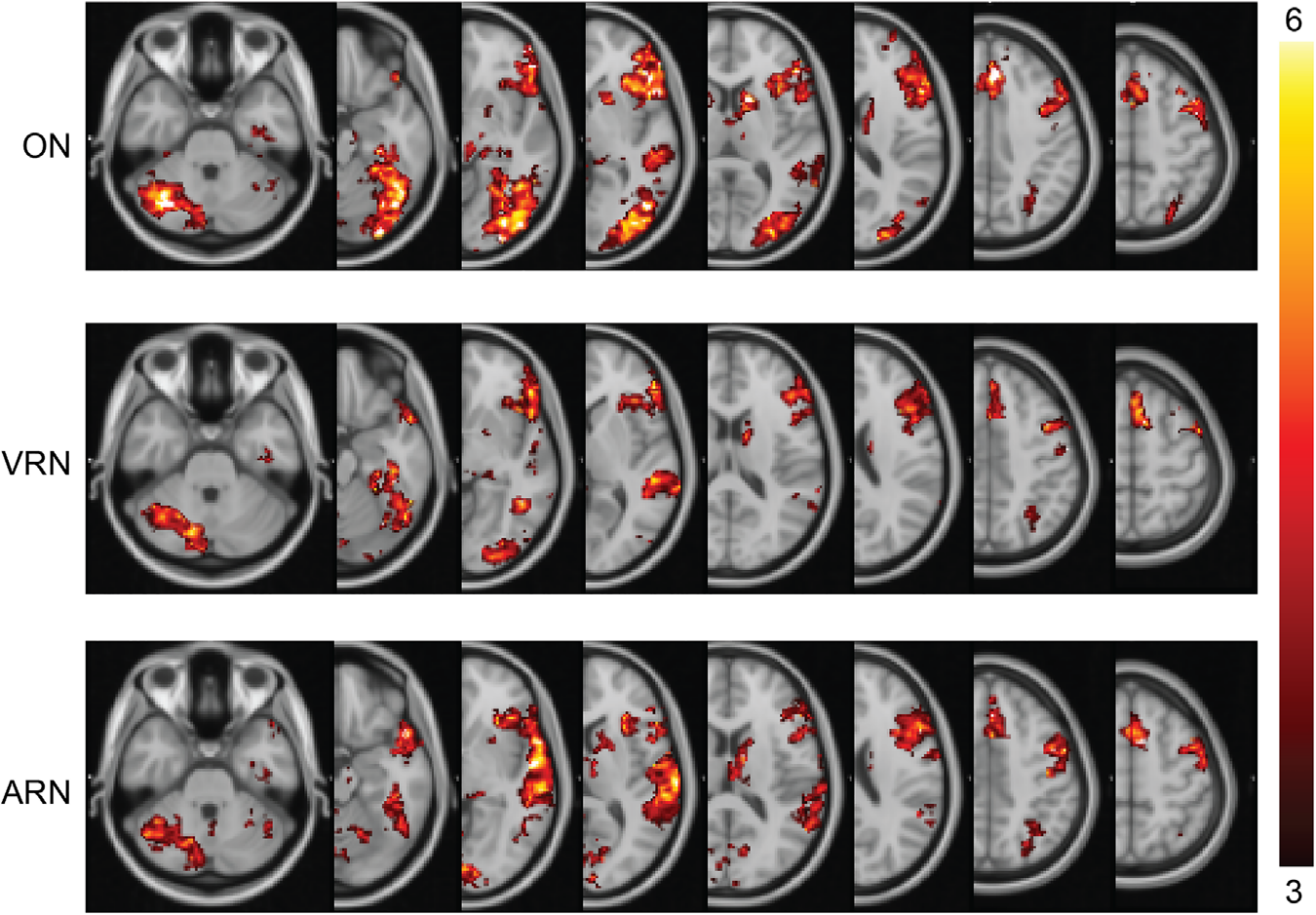
Mean statistical activation map of each task. The regions that were found activated in the group analysis of each task separately. Images are in radiological orientation. ON: Object Naming. VRN: Verbal Response Naming. ARN: Auditory Response Naming

To evaluate areas that were activated at the group level, standard second‐level analyses were completed and their conjunction was taken. This differs from the single subject results by identifying areas that may be significantly active at the group level even if they were not activated in each subject individually. It is evident in Figure 3 that all six language critical regions were reproduced successfully in the left hemisphere. Small activations in Broca's and Wernicke's areas in right hemisphere as well as a region in the left hemisphere in thalamus/caudate and in the right hemisphere of cerebellum were also found. In Table 2, the statistics of the conjunction map are reported. In Figure 4, the group maps for each task are presented for comparative reasons.

**TABLE 2:** The volume in mm^3^ and the percentage of activations in the conjunction map of the three tasks

	Region	Left hemisphere	Right hemisphere
Six language critical regions	Broca's area	13,264 (39.76%)	54 (0.16%)
	Wernicke's area	4520 (13.55%)	312 (0.94%)
	Exner's area	5136 (15.40%)	–
	Basal temporal	4760 (14.27%)	–
	Angular gyrus	696 (2.09%)	–
	SM area	4616 (13.84%)	–
	SUM	32,992 (98.90%)	366 (1.10%)
Other	Cerebellum	–	7904
	Thalamus/Caudate	296	–

*Note*: Only the six language critical regions were taken into account for the extraction of the percentages.

Abbreviation: SM area, supplementary motor area.

#### 3.2.1 Object naming task

In the group analysis map of ON, apart from the six language critical regions in the left hemisphere, a small cluster in right Broca's area and another small cluster in Wernicke's area were evident. Furthermore, a cluster was revealed in the left thalamus at the beginning of the optic radiation tract. In addition, an extensive portion of the visual cortex was activated that included all visual systems from V3 to V6 as well as lingual gyri. A considerable portion of the left caudate nuclei with little extent to the left putamen as well as a small cluster in the right caudate nuclei also survived thresholding.

#### 3.2.2 Verbal responsive naming task

In the group analysis map of VRN, similarly with the ON map, small clusters in the right Broca's and Wernicke's areas were revealed. A smaller in size cluster when compared to that of the ON map was found in the left caudate with little extent in the putamen, while a similar cluster with ON map was found in left thalamus. No activations were observed in the right deep brain structures. Visual systems were found activated mainly in left hemisphere and to a lesser degree than ON. In the right occipital lobe, only a small cluster in V4 was activated.

#### 3.2.3 Auditory responsive naming task

In the group analysis of the ARN, the entire bilateral superior temporal gyri with considerable extent to the temporal poles were activated. Broca's area in the right hemisphere was also found in similar regions as the other two tasks but with an extent in the right insula. The same region that was found in the ON and in the VRN for the right Wernicke's area was also activated in this task; however, the cluster merged with the activations in the STG and could not be separated. On top of that, a small cluster in Exner's area in the right hemisphere was activated. Of note are the activations in the deep brain structures. Similar to ON, a big cluster in left caudate was found activated but no activation was evident in the right hemisphere. Two clusters, one in the left posterior‐inferior aspect of the thalamus where the acoustic radiation begins and one in the anterior‐superior aspect of the thalamus where the anterior thalamic radiation ends, survived the statistical thresholding. Activations in the bilateral V2 with some extent in the lingual gyri were also found.

### 3.3 Comparison maps

**FIGURE 5:**
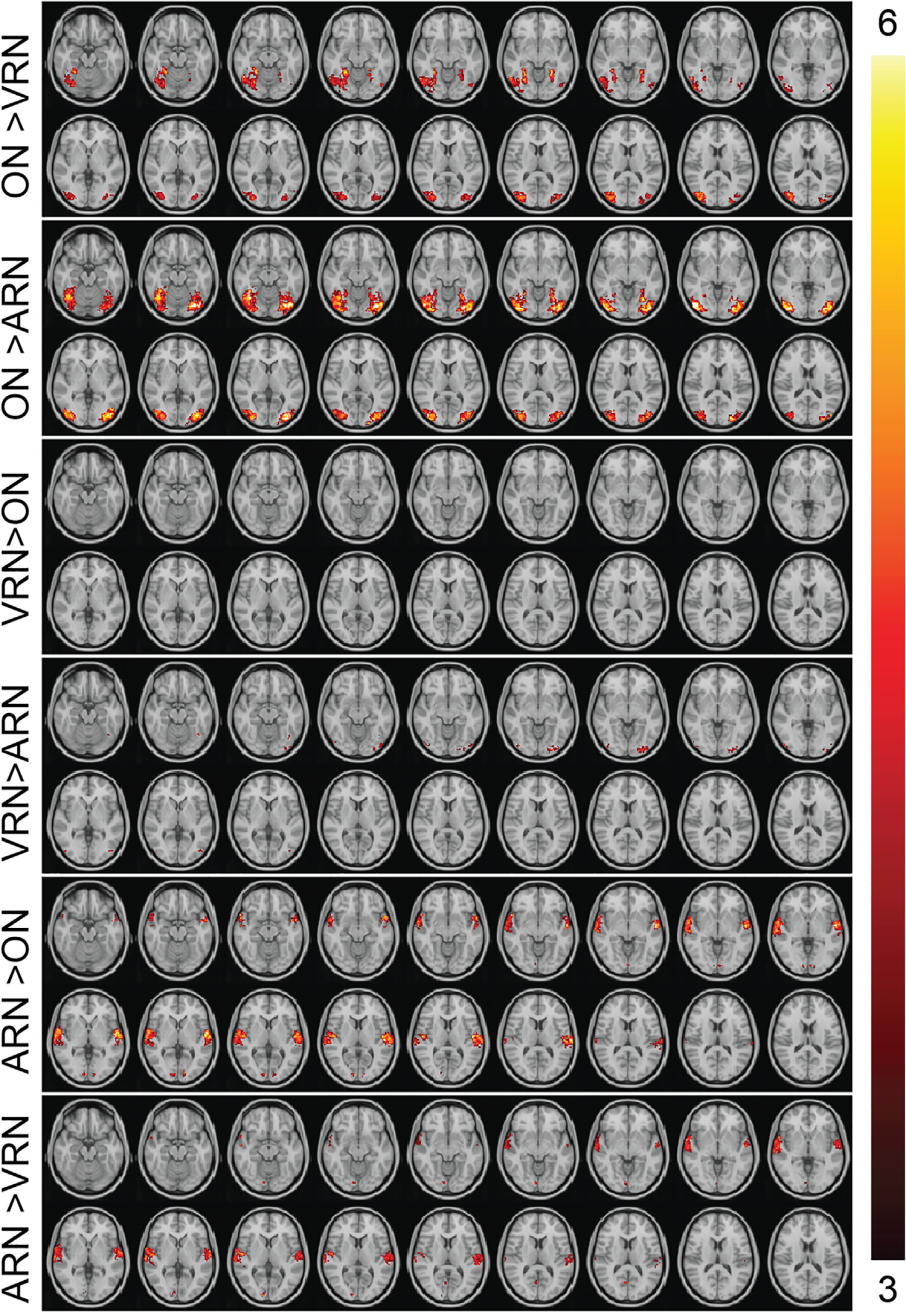
Comparison maps between all the pairs of the tasks. Images are in radiological orientation. ON: Object Naming. VRN: Verbal Response Naming. ARN: Auditory Response Naming

Furthermore, the comparison maps are presented in Figure 5. In these maps, the statistical differences between each pair of tasks are revealed resulting in a total of six different maps (ON > VRN, ON > ARN, VRN > ON, VRN > ARN, ARN > ON, and ARN > VRN). The regions activated in these maps can be considered as stimulus‐specific or stimulus‐decoding‐specific activations and future researchers may find evidence of their hypotheses/model in these maps.

When ON was compared to VRN (ON > VRN), it was found that during ON the visual systems V4 to V6 as well as lingual gyrus were more activated. All the visual systems, V3 to V6 as well as lingual gyri, that were found activated in the group analysis map of the ON were also found in the comparison of the ON with the ARN (ON > ARN). Furthermore, a small cluster in the SMA survived the statistical thresholding.

When VRN was compared to ON (VRN > ON), no cluster survived the statistical thresholding, while when VRN was compared with the ARN (VRN > ARN), two statistically significant clusters were found in occipital lobe: one in left V3 and V4 and one in the right V4.

When ARN was compared to ON (ARN > ON), the entire bilateral Heschl's gyri was relatively more activated as well as the superior part of the temporal poles as was found in the ARN group map. The bilateral V2 system activated in the ARN map was also found in this comparison map. In the comparison of the ARN with the VRN (ARN > VRN), the same clusters in the bilateral Heschl's gyri and the temporal poles were activated. Interestingly, in this comparison only the right V2 and a small cluster in the right lingual gyrus were found statistically significant and not in the left hemisphere. Noteworthy, some small clusters appeared in areas in which none of the tasks showed any activation, namely, (a) one in the junction of the precentral gyrus with the superior frontal and the middle frontal gyri in the left hemisphere, (b and c) two in the bilateral superior parietal gyri, (d) one in the most inferior part of the right superior parietal gyrus, in the junction of the supramarginal gyrus with the postcentral gyrus, and (e) one in the right precentral gyrus.

## 4 DISCUSSION

Language mapping using fMRI tasks has gained ground in the presurgical evaluation of epileptic patients being available in 96% of sites, while 60% of epileptic patients are receiving it prior to neurosurgery. At the same time, the golden standard noninvasive language lateralization test, namely, Wada test, is only available in as much as 76% of sites and only 43% of patients are receiving it (Benjamin, Li, et al., 2018). Even though this study has included sites worldwide, very few studies exist to date that verify the protocols being used in languages other than English.

To the best of our knowledge, this is the first study to standardize a protocol for language mapping in Greek, while at the same time, this protocol is able to reliably activate a language system wider than the Wernicke–Lichtheim–Geschwind model. Apart from the Greek native speakers, this may prove important for Greek bilingual speakers in other countries undertaking neurosurgery, as many studies suggest that language areas near tumors activate more reliably in the native language when compared to the later acquired languages (Kuper et al., 2021; Leung et al., 2020; Protopapas et al., 2016).

In this study, an fMRI protocol to map the language network in Greek native speakers has been adopted. The same protocol has been used to study language processing in epileptic patients who were English native speakers, showing that it can reliably activate six critical language regions, namely, Broca, Wernicke, Exner, SMA, BTLA, and AG areas (Benjamin et al., 2017). After translation, the same tasks were performed for the first time by healthy controls who were Greek native speakers and successfully reproduce similar activations. The tasks were ON (Bookheimer et al., 1995), VRN (Gaillard et al., 2002, 2001, 2004), and ARN (Bookheimer et al., 1997; Gaillard et al., 2004). We decided to perform the tasks in controls, free from any neurological disorders, in order to avoid any reorganization of language areas due to underlying pathologies. That way, we are able to standardize the protocol and provide evidence for the regions that are expected to be activated. The preprocessing that followed was minimal and in line with most common clinical practices in surgical planning (Benjamin, Dhingra, et al., 2018).

The conjunction map of the three tasks, that is, the areas that were found activated in all three tasks, is shown in Figure 3. It is obvious that all six language critical regions were activated in all group maps as well as an area in cerebellum and a small cluster between thalamus and caudate. These findings are in line with the original evaluation by Benjamin et al. (2017). Even if they did not discuss deep brain or cerebellum activations, these are evident in their Figures and they were confirmed after contacting the authors.

Even more interesting are the findings in the map of “appearances.” This map was created in order to assess the extent and border of activations that could be found in the subject‐specific level. This map is not a statistical map and should not be considered as one, though the information available in it may help to unravel the subject‐specific differences and variability that should be consider prior to neurosurgery. The intensity of the voxels in this map reveals the number of subjects having this particular voxel activated. We found high number of appearances in areas such as Broca, Wernicke, Exner, and SMA but smaller numbers in BTLA and AG. This can be interpreted as: while the first four regions are more consistent as to which area they cover, the latter, namely, BTLA and AG, are not fixed and their topography, borders, and activation extent vary significantly among individuals. This is in line with electrical stimulation studies of these areas and it is the first time shown so profoundly in an fMRI study (Enatsu et al., 2017; Mani et al., 2008; Ojemann, 1979; Roux et al., 2003; Steinmetz & Seitz, 1991).

The extent of each of the six language‐critical regions was different between the conjunction map and the map of “appearances.” In the conjunction map, Broca's area extended in pars triangularis, pars opercularis, pars orbitalis, anterior insula as well as a small region in the MFG adjusted to the IFG. In the map of “appearances,” few activations also extended to the inferior part of the precentral gyrus and the rostral MFG. We classified these areas as Broca's area even if they are in line with the “Broca's complex” for coherency purposes (Desai et al., 2006; Longe et al., 2007).

On the other hand, Wernicke's area was found very localized in the conjunction map in the posterior STG with little extension in the MTG and the adjacent supramarginal gyrus. In the map of “appearances,” the activations were more widely distributed covering areas in planum temporale, MTG, the posterior as well as the temporo‐occipital division and supramarginal gyrus even in the anterior division.

In most fMRI studies, the exact separation between Exner's area and Broca's area is somewhat arbitrary because the activations appear as a single big cluster. Such is the case in our study as well. We decided to classify the inferior part of the MFG that is adjacent to the IFG as Broca's area, while all the other areas more superior and/or more posterior to that were classified as Exner's area. In the conjunction analyses, activations were strictly limited to the posterior part of MFG, while very few activated voxels were found in the adjacent precentral gyrus. On the other hand, in the map of appearances, activations were evident in a wide extent of the MFG covering from the most inferior parts all the way to a small portion of the superior frontal gyrus. The maximum appearance of 14 out of 20 subjects occurred in the posterior part of the MFG adjacent to the precentral gyrus. Precentral gyrus was also found activated in a wider range in this map with four subjects showing activations even in the anterior part of the postcentral gyrus.

In the conjunction map, SMA was located mainly in the mesial aspect of the superior frontal gyrus with some extent in the subjacent paracingulate gyrus and little extent in the premotor cortex. In the map of “appearances,” the expansion in the premotor cortex and the cingulate gyrus was wider, while little extension was found toward more anterior regions.

For the AG, in the conjunction map, it was found as a small cluster in the intraparietal sulcus; however, in the map of “appearance,” a uniform dispersion in all directions was revealed. Activated areas were strictly in the superior part of the inferior parietal gyrus and the inferior aspect of the superior parietal gyrus. A maximum number of 8 appearances out of 20 subjects were found in the posterior part of the intraparietal sulcus. The fact that AG was evident in the group analysis of all tasks highlights that this area is not activated for a specific type of stimulus (visual or auditory). Furthermore, the fact that few appearances were found in the map of appearances and the dispersion was high suggests that this area can be found in a wide range of anatomical locations around intraparietal sulcus in the single subject level. This can explain, at least partially, why case studies and electrocortical studies do not converge for the symptoms being described (Roux et al., 2003).

BTLA was found in the conjunction map showing that it was activated independent of the stimuli in accordance with the literature (Antonucci et al., 2004; Kraft et al., 2014). The distance of the anterior border from the temporal tip was 3.7 cm located in the anterior part of the fusiform gyrus. The distance of its posterior border from the temporal tip was 10.2 cm located in the temporo‐occipital junction. Some extent in the adjacent ITG was also observed in the conjunction map and very little extent in the parahippocampal gyrus. Interestingly, in the map of “appearances,” the minimum and maximum distances for anterior and posterior borders from the temporal tip were very similar with that of the conjunction map at 3.5 and 10.6 cm, respectively. This can be attributed to it being extended from the most anterior part to the most posterior part of the fusiform gyrus at the temporo‐occipital junction in both maps. However, between‐subject variability was evident since the maximum value in the appearance map was found at 11 subjects. This is in line with the literature for this region reporting consistent between‐subject variability (Enatsu et al., 2017). The maximum value was observed in the fusiform gyrus, 8.1 cm from the temporal tip, while considerable extent is obvious in ITG as well as the parahippocampal gyrus.

Apart from each language critical region's extent, also of interest is the regions activated in each task separately as well as in comparison to each other as it may reveal task and/or stimuli‐specific areas of the brain. In the group analysis map of the ON, activations in the left thalamus that is located at the start of the optic radiation as well as activations in the occipital cortex can be attributed to the visual stimuli. All visual systems from V3 to V6 as well as lingual gyri were activated suggesting an extensive semantic processing of the exposed object. The V1 and V2 visual systems were not found activated as the control period successfully subtracted them, that is, they were also activated during the control period. Left caudate nuclei as well as a small portion of the left putamen and the right caudate survived the statistical thresholding. These finding are in line with previous studies suggesting activations in deep brain structures during language tasks, though their exact contribution is still a matter of debate (Alfredo et al., 2016b).

In the group analysis of the VRN, activations in the same cluster of thalami as in ON as well as the left occipital lobe and the V4 in the right occipital lobe can be attributed to the visual stimuli. Again, we believe that V1 and V2 were not activated as the control period subtracted them. A small cluster in caudate and putamen found activated in the left hemisphere.

In the ARN, the activations in the bilateral Heschl's gyri can be attributed to the acoustic stimuli. Curiously, activations in the bilateral V2 with some extent in the lingual gyri were also present that we could not explain apart from that the subjects were visualizing part of the task's auditory objects. Deep brain structures were also found activated in this map in the left hemisphere, with the activations in the caudate nuclei being the most profound, while two small clusters in the thalamus were evident.

In the comparison maps, when ON was compared to the VRN (ON > VRN), V3 and caudate nuclei were interestingly not found activated, meaning that they were activated during both tasks with no statistically significant intensity difference even if they did not appear activated in the group map of the VRN. On the other hand, when VRN was compared to the ON (VRN > ON), no clusters were found activated. These results reveal the similarity of these two tasks with respect to the language regions they recruit. At the same time, the difference in the comparison of the ON with the VRN reveals the extent of the visual system necessary to decode the objects in contrast to reading. Our hypothesis is that V1 and V2 are decoding the visual aspects of the stimuli in both tasks. Afterwards, for the ON task, the information flows toward the other visual systems, V3 to V6, to be further processed before being sent to the language system, while for the VRN the information flows from V1 and V2 directly to the language system, probably to the BTLA and/or AG.

To conclude, the aim of the study was the evaluation of a task‐based fMRI presurgical protocol for language mapping in Greek. Not only we verified that the same six language critical regions are activated in a different language than in its first evaluation, but we also revealed some activation in other crucial regions as well. Cerebellum has been previously described as activated in language tasks; however, its exact contribution to the language system is still a matter of debate (Alfredo et al., 2016b). In our study, we consistently found that the contralateral cerebellum was activated in all subjects as well as in the conjunction map of the group maps revealing that it does play a role that is not stimulus specific. Furthermore, deep brain structures such as caudate, thalamus, and putamen in the left hemisphere were also found activated. Even if their activations in the conjunction map were limited when we inspected the mean map for each task, we observed considerable activations especially in ON and ARN. In contrast, the lack of activations in these structures in the comparison maps shows that no statistically significant differences in activation between tasks exist. These results are contradicting to each other as to whether they are stimuli specific or not, revealing the complex mechanisms and role that these structures may play. Further studies with bigger cohorts and diverse tasks are necessary to unravel the contribution of deep brain structures to language production.

### 4.1 Limitations and future perspectives

There are some limitations in the current study that we need to pinpoint. First of all, the lack of neuropsychological evaluation of the subjects is a drawback as we could not correlate the activations found in a subject‐specific level with the performance in these tests. A methodological limitation is the conjunction map itself. The conjunction map may subtract the activations specific to stimuli; however, it also keeps the common activations in the language regions that may lead to decreased activation clusters in regions of interest. As a result, in most subjects the combination of two tasks, VRN and ARN or ON and ARN, was chosen from the two experts as the individual‐level language map considering that the activations were significantly decreased when using the conjunction of the three. Future studies may seek an alternative methodology to statistical analysis such as independent component analysis or multivoxel pattern analysis in order to overcome this drawback. Furthermore, even if it seems that a 1.5T scanner may be inadequate for clinical language mapping, the upgraded Siemens Avanto Fit scanner that was used is incorporating all equipment from a 3T Siemens Skyra model, except the magnetic field itself. Saying that, there may still be some drawbacks as the contrast that the 1.5T magnetic field offers is inferior when compared to that of a 3T field, though with less magnetic field inhomogeneities. Additionally, as we performed the tasks in healthy volunteers and we used different control period for the tasks than the English validated tasks, direct comparison of the activations could not be performed and differences in the maps cannot be attributed to the different language in which they were conducted. However, as the protocol is freely available (www.cogneuro.net/omfmri), future studies may seek to evaluate the same protocol in other languages as well as to perform comparison studies between different native languages to reveal probable differences in the processing of the language system.

## 5 CONCLUSION

To sum up, we successfully reproduced the appearance of the six language regions in Greek native speakers using a protocol that was previously evaluated in English native speakers. We were able not only to extract these in the group maps but, also, in all subjects’ single‐level maps demonstrating its efficacy for presurgical evaluation. The combination of three different stimulus modality tasks not only ensures that patients will cooperate to at least one, but also, if the patient cooperate in all, activations that solely belong to the language system will survive the conjunction map. This makes it ideal for the clinical practice where neurologists and neurosurgeons will need to take into account the language system and not activations to specific stimuli. Experienced clinicians, using the methodology originally proposed by Benjamin et al. (2017), should also be able to obtain within‐hemisphere localization using Greek, in addition to extracting language laterality.

## CONFLICT OF INTEREST

The authors declare no conflict of interest.

### PEER REVIEW

The peer review history for this article is available at https://publons.com/publon/10.1002/brb3.2609.

## Data Availability

The fMRI language protocol that was used is freely available at www.cogneuro.net/omfmri.
